# A mathematical model of vascular and hemodynamics changes in early and late forms of preeclampsia

**DOI:** 10.14814/phy2.15661

**Published:** 2023-04-26

**Authors:** Farbod Sedaghati, Rudolph L. Gleason

**Affiliations:** ^1^ The George W. Woodruff School of Mechanical Engineering Georgia Institute of Technology Atlanta Georgia USA; ^2^ The Wallace H. Coulter Department of Biomedical Engineering Georgia Institute of Technology Atlanta Georgia USA

**Keywords:** biomechanics, preeclampsia, pulsatility index, pulsewave model, uterine artery

## Abstract

Preeclampsia–eclampsia syndrome is a leading cause of maternal mortality. The precise etiology of preeclampsia is still not well‐defined and different forms exist, including *early* and *late* forms or preeclampsia, which may arise via distinctly different mechanisms. Low‐dose aspirin administered at the end of the first trimester in women identified as high risk has been shown to reduce the incidence of early, but not late, preeclampsia; however, current risk factors show only fair predictive capability. There is a pressing need to develop accurate descriptions for the different forms of preeclampsia. This paper presents 1D fluid, solid, growth, and remodeling models for pregnancies complicated with early and late forms of preeclampsia. Simulations affirm a broad set of literature results that early forms of preeclampsia are characterized by elevated uterine artery pulsatility index (UA‐PI) and total peripheral resistance (TPR) and lower cardiac output (CO), with modestly increased mean arterial blood pressure (MAP) in the first half of pregnancy, with elevation of TPR and MAP beginning at 20 weeks. Conversely, late forms of preeclampsia are characterized by only slightly elevated UA‐PI and normal pre‐term TPR, and slightly elevated MAP and CO throughout pregnancy, with increased TPR and MAP beginning after 34 weeks. Results suggest that preexisting arterial stiffness may be elevated in women that develop both early forms and late forms of preeclampsia; however, data that verify these results are lacking in the literature. Pulse wave velocity increases in early‐ and late‐preeclampsia, coincident with increases in blood pressure; however, these increases are mainly due to the strain‐stiffening response of larger arteries, rather than arterial remodeling‐derived changes in material properties. These simulations affirm that early forms of preeclampsia may be associated with abnormal placentation, whereas late forms may be more closely associated with preexisting maternal cardiovascular factors; simulations also highlight several critical gaps in available data.

## Graphical abstract

Pulsatility index and centerline blood velocity profiles with normal pregnancy and early and late preeclampsia.

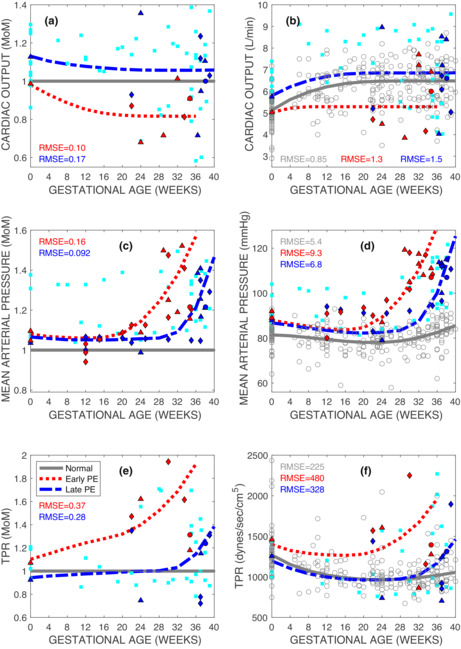

## 1 INTRODUCTION

Preeclampsia–eclampsia syndrome is the second leading cause of maternal mortality, is seven times more prevalent in developing versus developed nations, is 60% higher in Black versus White women in the United States, and is higher in women from rural versus urban in the United States and worldwide (Cameron et al., 2022; Fingar et al., 2017; Gaym et al., 2011; Osungbade & Ige, 2011). Preeclampsia is a multisystem disorder diagnosed as new‐onset hypertension after 20 weeks of gestation, combined with new‐onset proteinuria, thrombocytopenia, renal insufficiency, impaired liver function, pulmonary edema, or cerebral or visual symptoms. Months before the onset of preeclampsia, changes in maternal hemodynamics are often apparent, including altered uterine artery velocity waveforms (Fleischer et al., 1986; Kienast et al., 2016; Kumar et al., 2016; Papageorghiou & Leslie, 2007). Hemodynamic metrics, such as uterine artery pulsitility index (UA‐PI) and the presence of an early diastolic notch in the centerline velocity profile at 11 to 14 weeks of gestation, have shown to be useful in early assessment of risk of preeclampsia and fetal growth restriction. Furthermore, once identified as high risk, interventions such as early administration of low‐dose aspirin have been shown to prevent or reduce the severity of some forms of preeclampsia (Rolnik et al., 2017).

The precise etiology of preeclampsia is still not well‐defined. It is now widely recognized that different forms of preeclampsia may exist, including early and late forms of preeclampsia, which arise via distinctly different mechanisms (Tay et al., 2018; Valensise et al., 2008a). There are multiple definitions used to distinguish between early versus late forms of preeclampsia, based either on the gestational age at onset of preeclampsia or on the gestational age at delivery. Many define *early onset* versus *late onset* preeclampsia as the onset of preeclampsia before or after 34 weeks of gestation (Audibert et al., 2010; Browne et al., 2011a; Cong et al., 2015; Farina et al., 2011; Ferrazzi et al., 2018; Gómez‐Arriaga et al., 2014; Gyselaers et al., 2014; Lamale‐Smith et al., 2020; Robb et al., 2009; Valensise et al., 2008a); others use onset before or after 37 weeks of gestation (Demers et al., 2018; Perry et al., 2019). Other groups distinguish early versus late forms of preeclampsia based on the date of delivery, either before or after 34 weeks (Akolekar et al., 2011; Crovetto et al., 2015; Kaihura et al., 2009; Leite et al., 2019; Odibo et al., 2011; Oliveira et al., 2014; Onwudiwe et al., 2008; Park et al., 2013; Parra‐Cordero et al., 2013; Plasencia et al., 2015a; Poon, Kametas, et al., 2009; Poon, Karagiannis, et al., 2009; Poon, Maiz, et al., 2009; Scazzocchio et al., 2013; Stampalija et al., 2017) or 37 weeks (Akolekar et al., 2011; Buddeberg et al., 2018; Khalil et al., 2014; Leite et al., 2019; Melchiorre et al., 2011; Melchiorre et al., 2013; Melchiorre, Sutherland, Liberati, & Thilaganathan, 2012; Melchiorre, Sutherland, Watt‐Coote, et al., 2012). Given the scarcity of data on different forms of preeclampsia, we pooled data from different papers, using these different definitions, as “early forms” and “late forms” of preeclampsia, acknowledging that differences arise depending on the specific definition used.

Early forms of preeclampsia arise primarily from abnormal placentation and/or utero‐placental dysfunction and is characterized by elevated UA‐PI, lower cardiac output, and elevated total peripheral resistance (TPR), compared with uncomplicated pregnancies; blood pressure is typically higher throughout gestation in early‐onset preeclampsia, but increases rapidly in the second half of pregnancy leading to hypertension between 20 and 34 weeks of gestation. Early forms of preeclampsia are generally the more severe form of preeclampsia, are often associated with fetal growth restriction, and often require preterm delivery. Late forms of preeclampsia may arise from preexisting maternal factors, such as subclinical drivers of cardiovascular disease; for example, endothelial dysfunction or elevated arterial stiffness. Late forms of preeclampsia are characterized by slightly elevated UA‐PI, slightly higher cardiac output, and normal pre‐term TPR; hypertension arises at term. While the *early, placental‐forms* and the *late, maternal‐forms* of preeclampsia may, primarily, be driven via distinct mechanisms, maternal and utero‐placental risk factors can arise simultaneously and may synergistically contribute to the development, progression, timing of onset, and severity of preeclampsia.

The multifactorial nature of this condition renders risk prediction challenging. Hemodynamic indicators (e.g., UA‐PI) and circulating biomarkers at 11–14 weeks have been shown to yield some prognostic value in prediction of subsequent development of preeclampsia; however, these indicators show only fair prognostic accuracy of prediction of early forms of preeclampsia and may be even less predictive of late forms of preeclampsia (de Kat et al., 2019). Furthermore, metrics such as cardiac output, TPR, and UA‐PI require high‐resource imaging modalities and trained healthcare personnel (e.g., obstetric Doppler ultrasound, echocardiography). While these tools are nearly universally available in high‐resource settings, most mothers living in low‐resource settings, where much of the burden of preeclampsia exists, do not have access to equipment and trained personnel to perform these techniques. To improve preeclampsia prevention and reduce health inequities, there is a pressing need to develop a more accurate description and to develop more robust, resource‐appropriate, equitable risk assessment tools, for the early identification of risk of the different forms of preeclampsia, so that early prevention methods can be implemented in high‐risk mothers.

One‐dimensional (1D) hemodynamics models of the arterial tree can provide accurate estimates of the pressure, flow, and diameter wave propagation throughout the arterial system (Reymond et al., 2009). A validated 1D hemodynamics model that includes the growth and remodeling of the vascular and hemodynamic changes across gestation, comparing healthy pregnancy with early and late forms of preeclampsia, may provide a useful tool to better understand the development and progression of these complications and may reveal characteristics for improved early assessment of risk. We previously reported a mathematical framework of maternal vascular growth and remodeling and changes in hemodynamics in uncomplicated pregnancy (Gleason Jr. & Sedaghati, 2022). The purpose of this paper was to develop models that characterize early and late forms of preeclampsia, validate these models based on available results from the literature, and compare vascular and hemodynamic features across these illustrative simulations. We adapted a validated 1D model of the human vascular tree from the literature and prescribed inlet blood flow waveforms at the ascending aorta at 4‐week increments from 0 to 40 weeks of gestation. Peripheral resistances of each terminal vessel were adjusted to achieve mean arterial pressure and target flow rates at key, clinically accessible vascular sites at each gestational age. Vessel growth was governed by wall shear stress (and axial lengthening in uterine vessels) and changes in vessel distensibility was modeled as a function of vessel growth. Illustrative results are compared with a broad set of literature results.

## 2 METHODS

### 2.1 One‐dimensional modeling theoretical framework

#### 2.1.1 Balance relations

The arterial network was modeled as a tapered, elastic tubes in a one‐dimensional pulsatile fluid flow framework. Conservation of mass and the balance of linear momentum require that,

(1) $$\begin{array}{c} \frac{\partial A\left(z,t\right)}{\partial P\left(z,t\right)}\frac{\partial P\left(z,t\right)}{\partial t}+\frac{\partial Q\left(z,t\right)}{\partial z}=0\;\\ \text{and}\;\frac{\rho }{A}\frac{\partial Q}{\partial t}+\frac{\partial P}{\mathrm{dz}}=\frac{f}{A}-\frac{\rho }{A}\frac{\partial }{\partial z}\left(\frac{Q{\left(z,t\right)}^{2}}{A\left(z,t\right)}\right) \end{array}$$

where $A\left(z,t\right)$ is the instantaneous arterial lumen cross‐sectional area, $P\left(z,t\right)$ is the luminal pressure, and $Q\left(z,t\right)$ is the volumetric flow rate; z is the direction along the vessel axis, t is time, ρ is the blood density, f is the frictional force per unit length. We prescribe the velocity profile, $u_{z}\left(r,z,t\right)$, at any instant t and location z as

(2) $$u_{z}\left(r,z,t\right)=\bar{u}\left(z,t\right)\frac{\zeta +2}{\zeta }\left[1-{\left(\frac{r}{r_{i}}\right)}^{\zeta }\right]$$

where $\bar{u}\left(z,t\right)=Q\left(z,t\right)/A\left(z,t\right)$ is the mean velocity, ζ is a constant that governs the shape of the velocity profile, r is the radial coordinate, and $r_{i}$ is the lumen radius. Using the Naiver‐Stokes equations and integrating Equation (2),

(3) $$f=-2\left(\zeta +2\right)\mu \pi \frac{Q\left(z,t\right)}{A\left(z,t\right)},$$

where μ is the apparent blood viscosity. Given that all vessel diameters are above 250 microns the Fahraeus–Lindqvist effects are negligible and we assumed a constant value for μ = 4 mPa‐s.

#### 2.1.2 Solid mechanics

For the nongravid maternal vasculature, we let the reference distensibility $D_{\mathrm{ref}}$ at ($P_{\mathrm{ref}}$ = 100 mm Hg) be defined through the empirical relationship,

(4) $$D_{\mathrm{ref}}\left(\bar{d},P_{\mathrm{ref}}\right)=\frac{1}{\rho {\left(a_{2}/{\bar{d}}^{b_{2}}\right)}^{2}}$$

where $\bar{d}$ is the reference diameter, and $a_{2}$ and $b_{2}$ are parameters (Reymond et al., 2009). To account for the strain‐stiffening response of arteries, the distensibility at any pressure $D\left(P\right)$ was calculated as

(5) $$D\left(P\right)=\left[a_{1}+\frac{b_{1}}{1+{\left(\frac{P-P_{\max C}}{P_{\text{width}}}\right)}^{2}}\right]D_{\mathrm{ref}}$$

where $a_{1}$=0.4, $b_{1}$=5, $P_{\max C}$=2.67 kPa, and $P_{\text{width}}$= 4.0 kPa (Reymond et al., 2009). The area compliance $C_{A}$ is related to the distensibility as $C_{A}$=DA, which can be integrated to yield an expression for A versus *P*, as

(6) $$\begin{array}{c} A\left(P\right)=A_{\mathrm{ref}}\exp\left\{a_{1}D_{\mathrm{ref}}\left(P-P_{\mathrm{ref}}\right)+b_{1}D_{\mathrm{ref}}P_{\text{width}}\right.\\ \left.\left(\tan^{-1}\left(\frac{P-P_{\max C}}{P_{\text{width}}}\right)-\tan^{-1}\left(\frac{P_{\mathrm{ref}}-P_{\max C}}{P_{\text{width}}}\right)\right)\right\} \end{array}$$

#### 2.1.3 Boundary conditions at junctions

At bifurcations, we impose the continuity of flow and dynamic pressure at each branching point, neglecting minor pressure losses that may occur in the vicinity of the bifurcation. Thus, we let

(7) $$\sum_{i=1}^{N}Q_{i}\left(z,t\right)=0$$

(8) $$P_{i}\left(z,t\right)+\frac{\rho }{2}{\bar{u}}_{i}{\left(z,t\right)}^{2}=P_{j}\left(z,t\right)+\frac{\rho }{2}{\bar{u}}_{j}{\left(z,t\right)}^{2}\;i,j=1,2,\ldots ,N$$

where i and j denote different vessels of the junction and N represents the total number of branches at the junction.

#### 2.1.4 Terminal boundary conditions

To capture the resistance and compliance of the peripheral vasculature, beyond the terminal artery branches, a three‐element Windkessel (WK3) model is applied, which accounts for the proximal resistance ($R_{1}$), compliance (C), and distal resistance ($R_{2})$, where the total terminal resistance $R_{T}=R_{1}+R_{2}$. We let $R_{1}=Z_{c}$, where $Z_{c}$ is the characteristic impedance of the last arterial segment proximal to the terminal WK3 and, following Reymond et al. (2009), we let $Z_{c}=\sqrt{\rho }/\left(A_{\mathrm{ref}}\sqrt{D_{\mathrm{ref}}}\right)$, with values constrained to be within 5–40% of $R_{T}$ (Reymond et al., 2009).

### 2.2 Vasculature growth & remodeling throughout gestation

**FIGURE 1:**
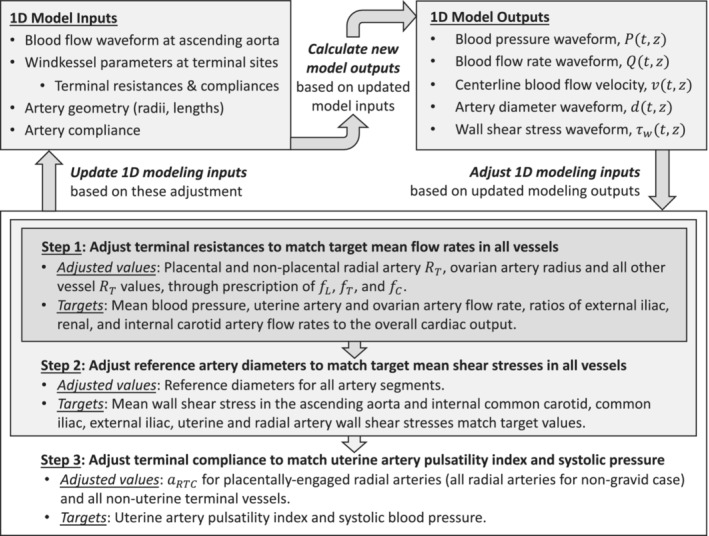
Method for solving for 1D FSGR model parameters. Given a set of modeling inputs, the modeling outputs are determined. Modeling outputs are compared with target values, defined at specific locations in the arterial tree at each gestational age, and the modeling inputs are adjusted. To aid in convergence, the iteration and parameter adjustment was performed in three steps. Step 1 was to adjust the terminal resistances until the flow rate at target locations and MAP converged (e.g., within 2% of target values). When the Step 1 criterion were met, Step 2 was to adjust the reference diameters of all vessels in network (while continuing to adjust the terminal resistances), until the target mean shear stress values in all arterial segments (and flow rate at target locations and MAP) converged. When the Step 1 and Step 2 criterion are met, Step 3 was to adjust the terminal compliances to match the UA‐PI and SBP, until all target values converged.

We employed the vascular network reported by Gleason and Sedaghati, 2022 (Gleason Jr. & Sedaghati, 2022), which was adapted from that of Reymond et al. (2009). A 1D pulse wave model of the human vasculature requires the following inputs: (*i*) the inlet blood flow waveform, (*ii*) the terminal Windkessel parameters, (*iii*) the radius and length of each artery segment, and (*iv*) the material properties of each artery segment (Figure 1). Given a set of modeling inputs, the modeling outputs are determined, namely $P\left(z,t\right)$, $Q\left(z,t\right)$, and $A\left(z,t\right)$ at every location in the arterial network, from which centerline blood flow velocity, $u_{z}\left(r=0,z,t\right)$, artery diameter waveforms, $d\left(z,t\right)$, and wall shear stress waveforms, $\tau_{w}\left(z,t\right)$, may be calculated. An implicit finite difference scheme was devised to solve the governing equations with the input parameters; see Gleason Jr. & Sedaghati, 2022. We discretized the time domain into $n_{t}$=500 time steps per cardiac cycle, with $\mathrm{dt}=60/\left(\mathrm{HR}*n_{t}\right)$. We let dz = 0.5 cm; for vessels with length segment length< 0.75 cm, we let $\mathrm{dz}=\left(\text{segment length}\right)/2$, to ensure that there are at least two elements for all vessel segments. Solutions typically converged after iterations through five cardiac cycles, but all results are shown for the 10th cardiac cycle. Modeling outputs are compared with target values, defined at specific locations in the arterial tree at each gestational age as described further, below, and the modeling inputs are adjusted. This loop is iterated until the targets are reached. All computations were performed using custom codes in Matlab^©^ 2021a (Mathworks).

#### 2.2.1 The inlet blood flow waveform

To establish the inlet blood flow waveform we let

(9) $$Q_{\text{inlet}}\left(t\right)=\mathrm{CO}\int_{0}^{t_{c}}q\left(t\right)\mathrm{dt}$$

where CO is the prescribed cardiac output, $t_{c}\left(\sec\right)=60/\mathrm{HR}$, HR is the prescribed heartrate in beats per minute (bpm), $q\left(t\right)$ describes the shape of the inlet flow waveform, where $\int_{0}^{t_{c}}q\left(t\right)\mathrm{dt}=1$. We digitized the mean of the inlet blood flow waveform presented in Reymond et al., (2009, Figure 4, panel A, upper curve) to determine $q\left(t\right)$. Changes in CO and HR throughout gestation were prescribed by fitting a representative curve to a broad set of results from the literature (Abduljalil et al., 2012; Bamfo et al., 2008; Bamfo, Kametas, Chambers, & Nicolaides, 2007; Bamfo, Kametas, Nicolaides, & Chambers, 2007; Borghi et al., 2000; Bosio et al., 1999; Buddeberg et al., 2018; Çağlar et al., 2016; Chapman et al., 1998; Clapp III & Capeless, 1997; Clark et al., 1989; Cong et al., 2015; Cornette et al., 2011; Del Bene et al., 2001; Dennis et al., 2012; Desai et al., 2004; D'Silva et al., 2013; Duvekot et al., 1995; Easterling et al., 1990; Estensen et al., 2012; Ferrazzi et al., 2018; Flo et al., 2010; Foo et al., 2018; Garcia‐Gonzalez et al., 2020; Geva et al., 1997; Gilson et al., 1997; Gyselaers et al., 2014; Hale et al., 2009; Hennessy et al., 1996; Jia et al., 2010; Kuleva et al., 2011; Lof et al., 2005; Mabie et al., 1994; Mahendru et al., 2014; Mahendru et al., 2017; Meah et al., 2016; Melchiorre et al., 2011; Melchiorre et al., 2013; Melchiorre, Sutherland, Liberati, & Thilaganathan, 2012; Melchiorre, Sutherland, Watt‐Coote, et al., 2012; Mesa et al., 1999; Moertl et al., 2012; Mone et al., 1996; Nii et al., 2018; Novelli et al., 2012; Ogueh et al., 2009; Perry et al., 2019; Perry et al., 2020; Poppas et al., 1997; Rang et al., 2007; Rang et al., 2008; Robson et al., 1989; San‐Frutos et al., 2005; Savu et al., 2012; Schannwell et al., 2002; Sengupta et al., 2017; Simmons et al., 2002; Solanki & Maitra, 2011; Stott et al., 2017; TamÁS et al., 2007; Tay et al., 2018; Tyldum et al., 2012; Vaddamani et al., 2017; Valensise et al., 2000; Valensise et al., 2001; Valensise et al., 2006; Valensise et al., 2008a; Valensise et al., 2008b; van der Graaf et al., 2013; Vårtun et al., 2014; Vasapollo et al., 2008; Vinayagam et al., 2018; Vlahović‐Stipac et al., 2010; Wolfe et al., 1999; Yin et al., 2019; Yosefy et al., 2012; Yuan et al., 2006) and were used to adjust $t_{c}$ and the inlet blood flow waveform $Q_{\text{inlet}}\left(t\right)$.

#### 2.2.2 Terminal Windkessel parameters

We adjusted the terminal resistances $R_{T}$ for all terminal sites so that the MAP matches target values by fitting a representative curve to a broad set of results from the literature (Borghi et al., 2000; Bosio et al., 1999; Browne et al., 2011a; Buddeberg et al., 2018; Çağlar et al., 2016; Chapman et al., 1998; Clapp III & Capeless, 1997; Clark et al., 1989; Cong et al., 2015; Cornette et al., 2011; Del Bene et al., 2001; Dennis et al., 2012; Desai et al., 2004; D'Silva et al., 2013; Duvekot et al., 1995; Easterling et al., 1990; Estensen et al., 2012; Ferrazzi et al., 2018; Flo et al., 2010; Foo et al., 2018; Geva et al., 1997; Gilson et al., 1997; Gyselaers et al., 2014; Hennessy et al., 1996; Jia et al., 2010; Khalil et al., 2014; Kuleva et al., 2011; Lof et al., 2005; Mabie et al., 1994; Mahendru et al., 2014; Mahendru et al., 2017; Meah et al., 2016; Melchiorre et al., 2011; Melchiorre et al., 2013; Melchiorre, Sutherland, Liberati, & Thilaganathan, 2012; Melchiorre, Sutherland, Watt‐Coote, et al., 2012; Mesa et al., 1999; Moertl et al., 2012; Mone et al., 1996; Nevo et al., 2010; Novelli et al., 2012; Ogueh et al., 2009; Perry et al., 2019; Perry et al., 2020; Poppas et al., 1997; Rang et al., 2007; Rang et al., 2008; Robb et al., 2009; Robson et al., 1989; San‐Frutos et al., 2005; Savu et al., 2012; Schannwell et al., 2002; Sengupta et al., 2017; Simmons et al., 2002; Solanki & Maitra, 2011; Stott et al., 2017; TamÁS et al., 2007; Tyldum et al., 2012; Vaddamani et al., 2017; Valensise et al., 2000; Valensise et al., 2001; Valensise et al., 2006; Valensise et al., 2008a; van der Graaf et al., 2013; Vårtun et al., 2014; Vasapollo et al., 2008; Vlahović‐Stipac et al., 2010; Wilson et al., 2007; Yin et al., 2019; Yosefy et al., 2012; Yuan et al., 2006), and that the relative changes in flow rate through the common iliac, renal, and internal carotid arteries match, as closely as possible, trends reported in Gleason and Sedaghati, 2022 (Gleason Jr. & Sedaghati, 2022). To match these values, we altered the limb, trunk, and cerebral resistances by the factors, $f_{L}$, $f_{T}$, and $f_{C}$, respectively, where $f_{J}=R_{T}/R_{T}^{\mathrm{Rey}}$, where $R_{T}^{\mathrm{Rey}}$ are the terminal resistances from the Reymond et al. (2009) network and J=L,T,orC. The compliances of all terminal sites were found via the relation $R_{T}C_{T}=a_{\mathrm{RTC}}$, where $a_{\mathrm{RTC}}$ was the same value for all terminal vessels, except radial arteries. For the radial arteries of the nongravid uterine vasculature, $a_{\mathrm{RTC}}$ was adjusted for all radial arteries so that the UA‐PI reached target values. For the gravid uterine vasculature, $a_{\mathrm{RTC}}$ for placentally engaged radial arteries was adjusted so that the UA‐PI reached target values; $a_{\mathrm{RTC}}$ for nonplacentally engaged radial arteries was set to the value in nongravid radial arteries.

#### 2.2.3 The radius and length of each artery segment

The lengths of all artery segments were taken from Gleason and Sedaghati (Gleason Jr. & Sedaghati, 2022). For the vessel diameters, we let each vascular segment grow radially to restore the mean wall shear stress (${\bar{\tau }}_{w}=32\mu \bar{Q}/\left(\pi d^{3}\right)$) to a prescribed target values ${\bar{\tau }}_{w}^{T}$. The target wall shear stress values for the maternal vasculature were taken from Gleason and Sedaghati (Gleason Jr. & Sedaghati, 2022). We let ${\bar{\tau }}_{w}^{T}$ for all uterine artery segments follow a prescribed, nonlinear trajectory. We let ${\bar{\tau }}_{w}^{T}$ for the arcuate arteries equal 1.05‐times that of the uterine artery target value, and let ${\bar{\tau }}_{w}^{T}$ for the radial arteries equal 1.10‐times that of the uterine artery target value, following Gleason and Sedghati (Gleason Jr. & Sedaghati, 2022).

#### 2.2.4 The material properties of each artery segment

For the nongravid uterus, we use Equation (5) to calculate the distensibility of each vessel in the vascular network. Following Gleason and Sedghati (Gleason Jr. & Sedaghati, 2022), for the gravid vascular networks, we let

(10) $$D_{\mathrm{ref}}\left(s\right)=\frac{D_{\mathrm{ref}}\left(s\right)}{R_{o}}\left[1+\frac{k_{r3}}{1+\exp\left\{-k_{r1}\left(\lambda_{\theta }^{G}\left(s\right)\lambda_{z}^{G}\left(s\right)-k_{r2}\right)\right\}}\right]$$

where s denotes the gestational age, $\lambda_{\theta }^{G}\left(s\right)=d\left(s\right)/d\left(s=0\right)$ and $\lambda_{z}^{G}\left(s\right)=\ell \left(s\right)/\ell \left(s=0\right)$ are the fractional increases in diameter (d) and length (ℓ) from the nongravid vasculature (i.e., at s=0) and the gravid time point of interest, $k_{r}^{1}$, $k_{r}^{2}$, and $k_{r}^{3}$ are remodeling parameters, and $R_{o}$ is the value of the expression in the square brackets at $\lambda_{\theta }^{G}\left(s\right)\lambda_{z}^{G}\left(s\right)$ = 1. We let $k_{r1}$=2.2 and $k_{r2}$=1.7 and let $k_{r3}$ = −0.85.

### 2.3 Illustrative simulations

We consider three illustrative simulations, meant to capture the changes in the maternal vasculature in uncomplicated pregnancies and in early and late forms of preeclampsia. Whenever possible, modeling input targets were prescribed from, and modeling results were compared to, published results. Since normal values of hemodynamic parameters may be different across different studies, regions, and people groups, to characterize differences between uncomplicated pregnancies and pregnancies complicated with early or late preeclampsia, we calculated multiples of the mean (MoM) as the ratio of the reported value in early or late preeclampsia to the reported value from the normal control group from the same study. We sought literature results that distinguished outcomes between early versus late preeclampsia (Akolekar et al., 2011; Audibert et al., 2010; Browne et al., 2011a; Buddeberg et al., 2018; Cong et al., 2015; Crovetto et al., 2015; Demers et al., 2018; Farina et al., 2011; Ferrazzi et al., 2018; Gómez‐Arriaga et al., 2014; Gyselaers et al., 2014; Khalil et al., 2014; Lamale‐Smith et al., 2020; Leite et al., 2019; Melchiorre et al., 2011; Melchiorre et al., 2013; Melchiorre, Sutherland, Liberati, & Thilaganathan, 2012; Melchiorre, Sutherland, Watt‐Coote, et al., 2012; Odibo et al., 2011; Oliveira et al., 2014; Onwudiwe et al., 2008; Park et al., 2013; Parra‐Cordero et al., 2013; Perry et al., 2019; Plasencia et al., 2015a; Poon, Kametas, et al., 2009; Poon, Karagiannis, et al., 2009; Poon, Maiz, et al., 2009; Robb et al., 2009; Scazzocchio et al., 2013; Stampalija et al., 2017; Valensise et al., 2008a); however, studies often do not make this distinction (Adekanmi et al., 2019; Bamfo et al., 2008; Borghi et al., 2000; Bosio et al., 1999; Çağlar et al., 2016; Dennis et al., 2012; Easterling et al., 1990; Eser et al., 2011; Foo et al., 2018; Jia et al., 2010; Khalil et al., 2012; Kienast et al., 2016; Kumar et al., 2016; Myatt et al., 2012; Odibo et al., 2011; Prajapati & Maitra, 2013; Rang et al., 2008; Sagol et al., 1999a; San‐Frutos et al., 2005; Simmons et al., 2002; Solanki & Maitra, 2011; Tay et al., 2018; Tyldum et al., 2012; Vasapollo et al., 2008; Yu et al., 2008; Yuan et al., 2006). To estimate the time course of the hemodynamic parameters with early versus late preeclampsia, we only considered results that make the distinction between early versus late forms. We do, however, include results that do not make the distinction between early versus late forms when presenting simulation results. The goodness of fit of the time course of each parameter, compared with the available literature values, is assessed by calculating the root mean square error, $\text{RMSE}=\sqrt{\sum_{i=1}^{n}\left(x_{i}^{\text{data}}-x_{i}^{\text{model}}\right)/n}$, where $x_{i}^{\text{data}}$ are the literature values and $x_{i}^{\text{model}}$ the model values at the corresponding gestational age and n is the number of experimental data points.

## 3 ILLUSTRATIVE SIMULATIONS

The prescribed modeling parameters, the properties for the maternal vasculature for each illustrative simulation, at each gestational age, are provided (Data S1—Supporting Tables)

### 3.1 Cardiac output, blood pressure, and total peripheral resistance

**FIGURE 2:**
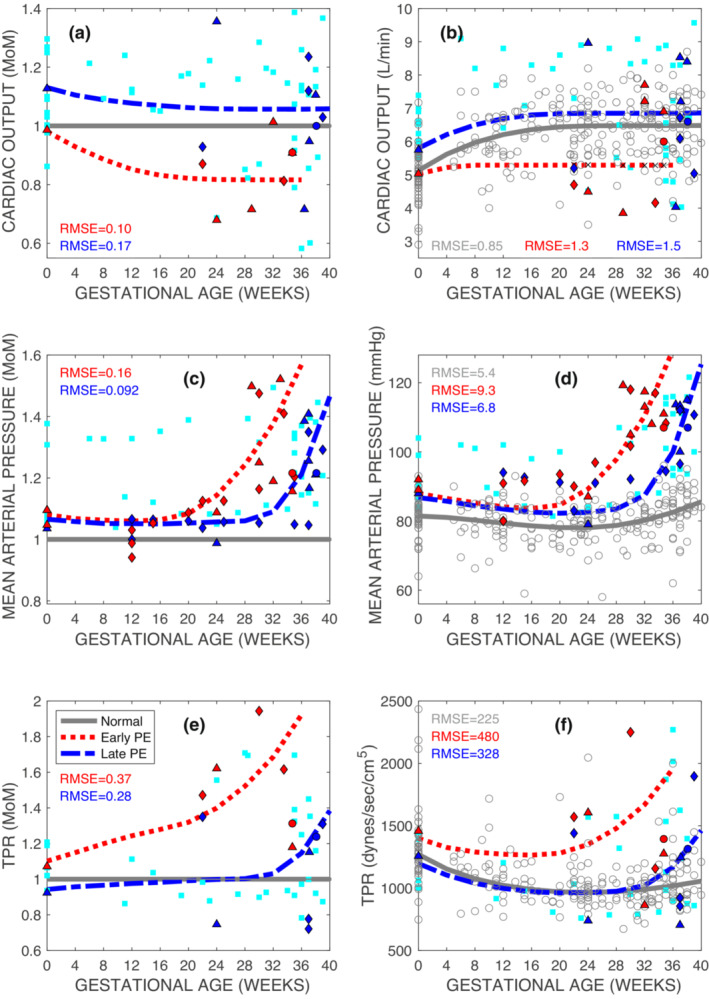
Cardiac output (CO, panel a and b), mean arterial blood pressure (MAP, panel c and d), and total peripheral resistance (TPR, panel e and f) presented both as multiples of the mean and true values versus gestational age for the *normal* (solid gray line), *early preeclampsia* (dashed red line), and *late preeclampsia* (blue dash‐dot line) illustrative simulations and for data reported in the literature. Data points were compiled from the literature for normal (gray open circles), early preeclampsia (red), and late preeclampsia (blue) (Abduljalil et al., 2012; Bamfo et al., 2008; Bamfo, Kametas, Chambers, & Nicolaides, 2007; Bamfo, Kametas, Nicolaides, & Chambers, 2007; Borghi et al., 2000; Bosio et al., 1999; Browne et al., 2011a; Buddeberg et al., 2018; Çağlar et al., 2016; Chapman et al., 1998; Clapp III & Capeless, 1997; Clark et al., 1989; Cong et al., 2015; Cornette et al., 2011; Del Bene et al., 2001; Dennis et al., 2012; Desai et al., 2004; D'Silva et al., 2013; Duvekot et al., 1995; Easterling et al., 1990; Estensen et al., 2012; Ferrazzi et al., 2018; Flo et al., 2010; Foo et al., 2018; Garcia‐Gonzalez et al., 2020; Geva et al., 1997; Gilson et al., 1997; Gyselaers et al., 2014; Hale et al., 2009; Hennessy et al., 1996; Jia et al., 2010; Khalil et al., 2014; Kuleva et al., 2011; Lof et al., 2005; Mabie et al., 1994; Mahendru et al., 2014; Mahendru et al., 2017; Meah et al., 2016; Melchiorre et al., 2011; Melchiorre et al., 2013; Melchiorre, Sutherland, Liberati, & Thilaganathan, 2012; Melchiorre, Sutherland, Watt‐Coote, et al., 2012; Mesa et al., 1999; Moertl et al., 2012; Mone et al., 1996; Nevo et al., 2010; Nii et al., 2018; Novelli et al., 2012; Ogueh et al., 2009; Perry et al., 2019; Perry et al., 2020; Poppas et al., 1997; Rang et al., 2007; Rang et al., 2008; Robb et al., 2009; Robson et al., 1989; San‐Frutos et al., 2005; Savu et al., 2012; Schannwell et al., 2002; Sengupta et al., 2017; Simmons et al., 2002; Solanki & Maitra, 2011; Stott et al., 2017; TamÁS et al., 2007; Tay et al., 2018; Tyldum et al., 2012; Vaddamani et al., 2017; Valensise et al., 2000; Valensise et al., 2001; Valensise et al., 2006; Valensise et al., 2008a; Valensise et al., 2008b; van der Graaf et al., 2013; Vårtun et al., 2014; Vasapollo et al., 2008; Vinayagam et al., 2018; Vlahović‐Stipac et al., 2010; Wilson et al., 2007; Wolfe et al., 1999; Yin et al., 2019; Yosefy et al., 2012; Yuan et al., 2006). For early and late preeclampsia, marker shapes indicate how each study distinguished between early versus late preeclampsia; namely, onset before or after 34 weeks (triangle markers) or 37 weeks (diamond markers) or delivery before or aftern37 weeks (circle markers). The cyan squares are data from studies that did not distinguish between early versus late preeclampsia and, therefore, combine data for these two groups. RMSE in gray, red, and blue indicate values for normal, early preeclampsia, and late preeclampsia, respectively.

Cardiac input (CO) increases by 20%–30% from conception to term in uncomplicated pregnancy (Abduljalil et al., 2012; Bamfo et al., 2008; Bamfo, Kametas, Chambers, & Nicolaides, 2007; Bamfo, Kametas, Nicolaides, & Chambers, 2007; Borghi et al., 2000; Bosio et al., 1999; Buddeberg et al., 2018; Çağlar et al., 2016; Chapman et al., 1998; Clapp III & Capeless, 1997; Clark et al., 1989; Cong et al., 2015; Cornette et al., 2011; Del Bene et al., 2001; Dennis et al., 2012; Desai et al., 2004; D'Silva et al., 2013; Duvekot et al., 1995; Easterling et al., 1990; Estensen et al., 2012; Ferrazzi et al., 2018; Flo et al., 2010; Foo et al., 2018; Garcia‐Gonzalez et al., 2020; Geva et al., 1997; Gilson et al., 1997; Gyselaers et al., 2014; Hale et al., 2009; Hennessy et al., 1996; Jia et al., 2010; Kuleva et al., 2011; Lof et al., 2005; Mabie et al., 1994; Mahendru et al., 2014; Mahendru et al., 2017; Meah et al., 2016; Melchiorre et al., 2011; Melchiorre et al., 2013; Melchiorre, Sutherland, Liberati, & Thilaganathan, 2012; Melchiorre, Sutherland, Watt‐Coote, et al., 2012; Mesa et al., 1999; Moertl et al., 2012; Mone et al., 1996; Nii et al., 2018; Novelli et al., 2012; Ogueh et al., 2009; Perry et al., 2019; Perry et al., 2020; Poppas et al., 1997; Rang et al., 2007; Rang et al., 2008; Robson et al., 1989; San‐Frutos et al., 2005; Savu et al., 2012; Schannwell et al., 2002; Sengupta et al., 2017; Simmons et al., 2002; Solanki & Maitra, 2011; Stott et al., 2017; TamÁS et al., 2007; Tay et al., 2018; Tyldum et al., 2012; Vaddamani et al., 2017; Valensise et al., 2000; Valensise et al., 2001; Valensise et al., 2006; Valensise et al., 2008a; Valensise et al., 2008b; van der Graaf et al., 2013; Vårtun et al., 2014; Vasapollo et al., 2008; Vinayagam et al., 2018; Vlahović‐Stipac et al., 2010; Wolfe et al., 1999; Yin et al., 2019; Yosefy et al., 2012; Yuan et al., 2006) (Figure 2). In early forms of preeclampsia, CO is generally lower, reaching ~80% of the CO of uncomplicated pregnancies beyond 20 weeks, whereas in late forms of preeclampsia, CO is ~5% higher than uncomplicated pregnancies beyond 20 weeks (Buddeberg et al., 2018; Cong et al., 2015; Ferrazzi et al., 2018; Gyselaers et al., 2014; Melchiorre et al., 2011; Melchiorre et al., 2013; Melchiorre, Sutherland, Liberati, & Thilaganathan, 2012; Perry et al., 2019; Vaddamani et al., 2017). Differences in CO may be due to changes in heart rate and stroke volume, which are both lower in early forms of preeclampsia compared with late forms of preeclampsia and uncomplicated pregnancies. Mean arterial pressure (MAP) is slightly higher in both early and late forms of preeclampsia, before 20 weeks and began to increase at ~20‐ and ~32‐weeks in early and late forms of preeclampsia, respectively (Borghi et al., 2000; Bosio et al., 1999; Browne et al., 2011a; Buddeberg et al., 2018; Çağlar et al., 2016; Chapman et al., 1998; Clapp III & Capeless, 1997; Clark et al., 1989; Cong et al., 2015; Cornette et al., 2011; Del Bene et al., 2001; Dennis et al., 2012; Desai et al., 2004; D'Silva et al., 2013; Duvekot et al., 1995; Easterling et al., 1990; Estensen et al., 2012; Ferrazzi et al., 2018; Flo et al., 2010; Foo et al., 2018; Geva et al., 1997; Gilson et al., 1997; Gyselaers et al., 2014; Hennessy et al., 1996; Jia et al., 2010; Khalil et al., 2014; Kuleva et al., 2011; Lof et al., 2005; Mabie et al., 1994; Mahendru et al., 2014; Mahendru et al., 2017; Meah et al., 2016; Melchiorre et al., 2011; Melchiorre et al., 2013; Melchiorre, Sutherland, Liberati, & Thilaganathan, 2012; Melchiorre, Sutherland, Watt‐Coote, et al., 2012; Mesa et al., 1999; Moertl et al., 2012; Mone et al., 1996; Nevo et al., 2010; Novelli et al., 2012; Ogueh et al., 2009; Perry et al., 2019; Perry et al., 2020; Poppas et al., 1997; Rang et al., 2007; Rang et al., 2008; Robb et al., 2009; Robson et al., 1989; San‐Frutos et al., 2005; Savu et al., 2012; Schannwell et al., 2002; Sengupta et al., 2017; Simmons et al., 2002; Solanki & Maitra, 2011; Stott et al., 2017; TamÁS et al., 2007; Tyldum et al., 2012; Vaddamani et al., 2017; Valensise et al., 2000; Valensise et al., 2001; Valensise et al., 2006; Valensise et al., 2008a; van der Graaf et al., 2013; Vårtun et al., 2014; Vasapollo et al., 2008; Vlahović‐Stipac et al., 2010; Wilson et al., 2007; Yin et al., 2019; Yosefy et al., 2012; Yuan et al., 2006). The total peripheral resistance (TPR) decreases in the first trimester, in all cases, but the TPR is higher in early forms, compared with normal and late forms of preeclampsia, throughout pregnancy (Bamfo et al., 2008; Bamfo, Kametas, Chambers, & Nicolaides, 2007; Bamfo, Kametas, Nicolaides, & Chambers, 2007; Borghi et al., 2000; Bosio et al., 1999; Browne et al., 2011a; Buddeberg et al., 2018; Çağlar et al., 2016; Chapman et al., 1998; Clapp III & Capeless, 1997; Clark et al., 1989; Cornette et al., 2011; Del Bene et al., 2001; Dennis et al., 2012; Desai et al., 2004; D'Silva et al., 2013; Duvekot et al., 1995; Easterling et al., 1990; Estensen et al., 2012; Ferrazzi et al., 2018; Flo et al., 2010; Foo et al., 2018; Garcia‐Gonzalez et al., 2020; Geva et al., 1997; Gilson et al., 1997; Hennessy et al., 1996; Jia et al., 2010; Kuleva et al., 2011; Lof et al., 2005; Mabie et al., 1994; Mahendru et al., 2014; Mahendru et al., 2017; Meah et al., 2016; Melchiorre et al., 2011; Melchiorre et al., 2013; Melchiorre, Sutherland, Liberati, & Thilaganathan, 2012; Melchiorre, Sutherland, Watt‐Coote, et al., 2012; Mesa et al., 1999; Novelli et al., 2012; Ogueh et al., 2009; Perry et al., 2019; Perry et al., 2020; Poppas et al., 1997; Robson et al., 1989; San‐Frutos et al., 2005; Savu et al., 2012; Schannwell et al., 2002; Sengupta et al., 2017; Simmons et al., 2002; Solanki & Maitra, 2011; Stott et al., 2017; Tay et al., 2018; Vaddamani et al., 2017; Valensise et al., 2000; Valensise et al., 2001; Valensise et al., 2006; Valensise et al., 2008a; van der Graaf et al., 2013; Vårtun et al., 2014; Vasapollo et al., 2008; Vinayagam et al., 2018; Vlahović‐Stipac et al., 2010). In early forms, TPR starts at 1.1‐fold higher at conception, 1.24‐fold higher at 12 weeks, and increases to 1.7‐fold higher 32 weeks. The TPR of normal and late forms of preeclampsia are similar until 32 weeks of gestation; after 32 weeks, the TPR increases in late forms of preeclampsia, reaching 1.14‐fold higher at 36 weeks.

### 3.2 Uterine artery pulsatility index and perfusion

**FIGURE 3:**
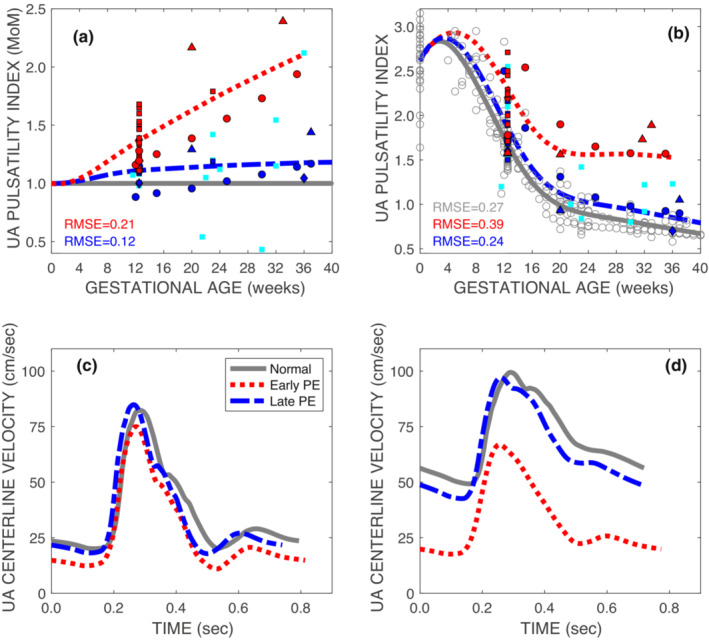
Uterine artery pulsatility index (panel a and b) and centerline blood flow velocity profiles over one cardiac cycle at 12 weeks (panel c) and 36 weeks (panel d) of gestation for *normal* (gray line), *early preeclampsia* (red dotted line), and *late preeclampsia* (blue dash‐dot line) illustrative simulations. Data points were compiled from the literature for normal (gray open circles), early preeclampsia (red), and late preeclampsia (blue) (Abdel Moety et al., 2016; Adekanmi et al., 2019; Akolekar et al., 2011; Arcangeli et al., 2013; Audibert et al., 2010; Bahlmann et al., 2012; Bamfo, Kametas, Chambers, & Nicolaides, 2007; Borna & Sci, 2015; Browne et al., 2011b; Carter et al., 2015; Cavoretto et al., 2020; Cheng et al., 2015; Coppens et al., 1996; Crovetto et al., 2015; D'Antonio et al., 2020; De Paco et al., 2014; Demers et al., 2018; Deurloo et al., 2009; Dickey et al., 1994; Drouin et al., 2018; Engmann et al., 1999; Ergin & Yayla, 2015; Eser et al., 2011; Everett et al., 2012; Farina et al., 2011; Fugino et al., 1993; García et al., 2016; Garcia‐Gonzalez et al., 2020; Gómez et al., 2005; Gómez et al., 2008; Gómez‐Arriaga et al., 2014; Guanes et al., 1996; Guedes‐Martins et al., 2014; Hanchard et al., 2020; Hsieh et al., 2000; Jamal et al., 2013; Jauniaux et al., 1991; Jurkovic et al., 1991; Kaminopetros et al., 1991; Khalil et al., 2012; Khalil et al., 2014; Khalil et al., 2016; Kienast et al., 2016; Kumar et al., 2016; Lai et al., 2013; Lamale‐Smith et al., 2020; Leite et al., 2019; Mäkikallio et al., 2004; Myatt et al., 2012; Odibo et al., 2011; Oliveira et al., 2014; Olofsson et al., 1993; Onwudiwe et al., 2008; Özkaya et al., 2007; Papageorghiou et al., 2001; Park et al., 2013; Parra‐Cordero et al., 2013; Parry et al., 2017; Perry et al., 2020; Pilalis et al., 2007; Plasencia et al., 2015b; Ponmozhi et al., 2019; Poon, Kametas, et al., 2009; Poon, Karagiannis, et al., 2009; Poon, Maiz, et al., 2009; Prajapati & Maitra, 2013; Prodan et al., 2019; Rigano et al., 2010; Sagol et al., 1999b; Scazzocchio et al., 2013; Stampalija et al., 2017; Tay et al., 2018; Taylor et al., 2020; Tekay et al., 1996; Tezcan et al., 2015; Valentin et al., 1996; Wang et al., 2010; Wilson et al., 2007; Yalti et al., 2003; Yu et al., 2008; Zaidi, 2009; Zebitay et al., 2016). For early and late preeclampsia marker shapes indicate how each study distinguished between early versus late preeclampsia; namely, onset before or after 34 weeks (triangle markers) or 37 weeks (diamond markers) or delivery before 34 weeks (square markers) or 37 weeks (circle markers) of gestation. The cyan squares are data from studies that did not distinguish between early versus late preeclampsia and, therefore, combine data for these two groups. RMSE in gray, red, and blue indicate values for normal, early preeclampsia, and late preeclampsia, respectively.

The UA‐PI is a noninvasive metric that correlates with reduction in peripheral resistance of the uterine vascular and, therefore, vascular perfusion. In uncomplicated pregnancies, UA‐PI dramatically decreases between 4 and 20 weeks of gestation, and values are tightly conserved across numerous studies (Abdel Moety et al., 2016; Adekanmi et al., 2019; Akolekar et al., 2011; Arcangeli et al., 2013; Audibert et al., 2010; Bahlmann et al., 2012; Bamfo, Kametas, Chambers, & Nicolaides, 2007; Borna & Sci, 2015; Browne et al., 2011b; Carter et al., 2015; Cavoretto et al., 2020; Cheng et al., 2015; Coppens et al., 1996; Crovetto et al., 2015; D'Antonio et al., 2020; De Paco et al., 2014; Demers et al., 2018; Deurloo et al., 2009; Dickey et al., 1994; Drouin et al., 2018; Engmann et al., 1999; Ergin & Yayla, 2015; Eser et al., 2011; Everett et al., 2012; Farina et al., 2011; Fugino et al., 1993; García et al., 2016; Garcia‐Gonzalez et al., 2020; Gómez et al., 2005; Gómez et al., 2008; Gómez‐Arriaga et al., 2014; Guanes et al., 1996; Guedes‐Martins et al., 2014; Hanchard et al., 2020; Hsieh et al., 2000; Jamal et al., 2013; Jauniaux et al., 1991; Jurkovic et al., 1991; Kaminopetros et al., 1991; Khalil et al., 2012; Khalil et al., 2014; Khalil et al., 2016; Kienast et al., 2016; Kumar et al., 2016; Lai et al., 2013; Lamale‐Smith et al., 2020; Leite et al., 2019; Mäkikallio et al., 2004; Myatt et al., 2012; Odibo et al., 2011; Oliveira et al., 2014; Olofsson et al., 1993; Onwudiwe et al., 2008; Özkaya et al., 2007; Papageorghiou et al., 2001; Park et al., 2013; Parra‐Cordero et al., 2013; Parry et al., 2017; Perry et al., 2020; Pilalis et al., 2007; Plasencia et al., 2015b; Ponmozhi et al., 2019; Poon, Kametas, et al., 2009; Poon, Karagiannis, et al., 2009; Prajapati & Maitra, 2013; Prodan et al., 2019; Rigano et al., 2010; Sagol et al., 1999b; Scazzocchio et al., 2013; Stampalija et al., 2017; Tay et al., 2018; Taylor et al., 2020; Tekay et al., 1996; Tezcan et al., 2015; Valentin et al., 1996; Wang et al., 2010; Wilson et al., 2007; Yalti et al., 2003; Yu et al., 2008; Zaidi, 2009; Zebitay et al., 2016) (Figure 3). UA‐PI may be slightly increased (~1.1‐ to ~1.2‐fold) between 12‐ and 32 weeks of gestation in late forms of preeclampsia, but are more dramatically increased (~1.4‐ to ~2.0‐fold) between 12‐ and 32 weeks in early forms of preeclampsia (Audibert et al., 2010; Farina et al., 2011; Lamale‐Smith et al., 2020; Demers et al., 2018; Onwudiwe et al., 2008; Poon, Kametas, et al., 2009; Poon, Karagiannis, et al., 2009; Poon, Maiz, et al., 2009; Akolekar et al., 2011; Odibo et al., 2011; Parra‐Cordero et al., 2013; Scazzocchio et al., 2013; Park et al., 2013; Oliveira et al., 2014; Crovetto et al., 2015; Plasencia et al., 2015a; Stampalija et al., 2017; Leite et al., 2019; Khalil et al., 2014; Browne et al., 2011b; Abdel Moety et al., 2016). The center‐line velocity of the uterine arteries show that, at 12 weeks of gestation, this differences in UA‐PI arise primarily via lower values of the diastolic velocity, as the peak systolic velocity is similar between the normal and early preeclampsia cases. At 32 weeks of gestation, differences in both the systolic and diastolic centerline velocity are apparent.

**FIGURE 4:**
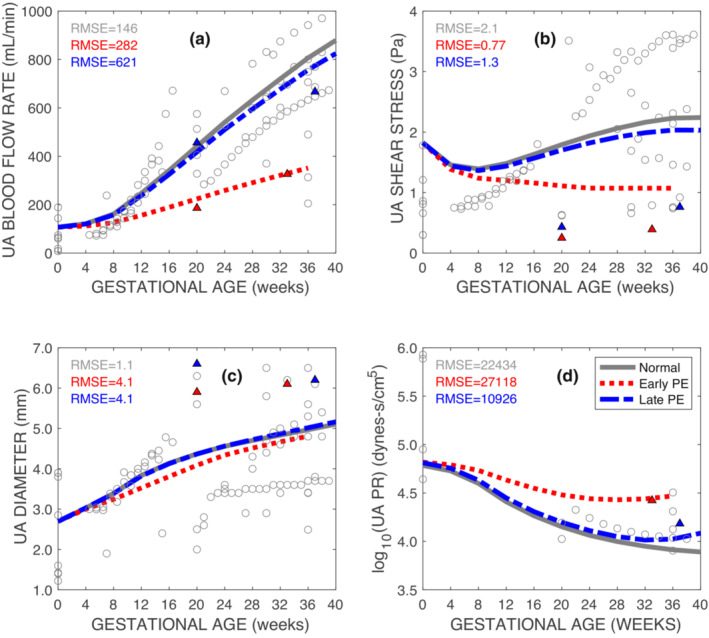
Uterine artery bilateral blood flow rate (panel a), calculated mean wall shear stress (panel b), diameter (panel c) and peripheral resistance (panel d) versus gestational age for *normal* (gray line), *early preeclampsia* (red dotted line) and *late preeclampsia* (blue dash‐dot line) illustrative simulations. Data points were compiled from the literature for normal (gray open circles), early preeclampsia (red), and late preeclampsia (blue) (Browne et al., 2011b; Dickey, 1995; Dickey et al., 1994; Dickey et al., 1995; Dickey & Hower, 1995; Flo et al., 2010; Jeffreys et al., 2006; Konje et al., 2001; Palmer et al., 1992; Thaler et al., 1990; Wilson et al., 2007; Zamudio et al., 1995). For early and late preeclampsia marker shapes indicate how each study distinguished between early versus late preeclampsia; namely, onset before or after 34 weeks (triangle markers) or 37 weeks (diamond markers) or delivery before or after 34 weeks (square markers) or 37 weeks (circle markers) of gestation. The cyan squares are data from studies that did not distinguish between early versus late preeclampsia and, therefore, combine data for these two groups. RMSE in gray, red, and blue indicate values for normal, early preeclampsia, and late preeclampsia, respectively.

Fewer studies are available that quantify the uterine artery (UA) blood flow rate and diameter in normal pregnancy and early and late forms of preeclampsia (Browne et al., 2011b; Dickey, 1995; Dickey et al., 1994; Dickey et al., 1995; Dickey & Hower, 1995; Flo et al., 2010; Jeffreys et al., 2006; Konje et al., 2001; Palmer et al., 1992; Thaler et al., 1990; Wilson et al., 2007; Zamudio et al., 1995) (Figure 4). Bilateral uterine artery blood flow increases by >8‐fold. Only two studies were found that report changes in UA blood flow and diameter in early and late forms of preeclampsia (Browne et al., 2011a; Lamale‐Smith et al., 2020). Browne et al. (Browne et al., 2011a) and Lamale‐Smith et al. (Lamale‐Smith et al., 2020) reported UA blood flow rates of 0.47 and 0.39 MoM in early forms of preeclampsia at 33 and 32 weeks of gestation and 0.87 and 0.96 MoM in late forms of preeclampsia at 37 and 32 weeks, respectively. Both studies reported UA diameters to be nearly equal for normal pregnancy and early and late forms of preeclampsia. Note that the normal values for uterine artery diameter from these studies were higher than the average values from other studies. As a result, the diameters from early and late forms of preeclampsia from these papers are also above the target values; however, since the MoM values were near 1.0 from these studies, the target diameters were prescribed to be similar to normal pregnancy. UA wall shear stress values, calculated from blood flow and diameter from these studies, were 0.49 and 0.48 MoM in early preeclampsia and 0.82 and 0.84 MoM in late preeclampsia, respectively. The peripheral resistance of the uterine vasculature decreased significantly in the first half of pregnancy, with similar values in late forms of preeclampsia and uncomplicated pregnancies, but decreases less in early forms of preeclampsia. These results are consistent with the changes in UA PI, which are a surrogate indicator of peripheral resistance in the uterine vasculature.

### 3.3 Arterial stiffness

**FIGURE 5:**
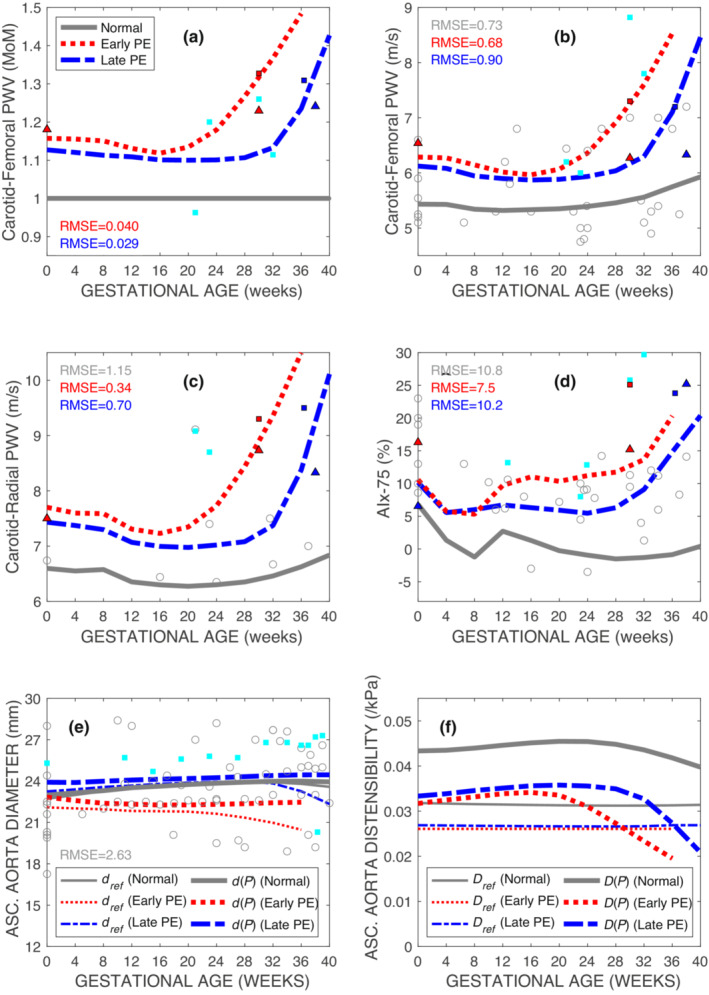
Carotid‐femoral pulse wave velocity (PWV, panel a and b), carotid‐radial PWV (panel c), augmentation index (${\mathrm{AI}}_{x}^{75}$, panel d), reference and in vivo ascending aorta diameter (panel E), and reference and in vivo ascending aorta distensibility versus gestational age for *normal* (gray line), *early preeclampsia* (red dotted line) and *late preeclampsia* (blue dash‐dot line) illustrative simulations. Data points were compiled from the literature for normal (gray open circles), early preeclampsia (red diamonds), and late preeclampsia (blue triangles) (Anastasakis et al., 2008; Callaghan et al., 2018; Everett et al., 2012; Franz et al., 2013; Kaihura et al., 2009; Khalil et al., 2012; Khalil et al., 2014; Lampinen et al., 2006; Mahendru et al., 2014; Mahendru et al., 2017; Rang et al., 2007; Robb et al., 2009; Rodriguez et al., 2018; Savvidou et al., 2011; Tay et al., 2018; van der Graaf et al., 2013). The cyan squares are data from studies that did not distinguish between early versus late preeclampsia and, therefore, combine data for these two groups. For early and late preeclampsia, marker shapes indicate how each study distinguished between early versus late preeclampsia; namely, onset before or after 34 weeks (triangle markers) or delivery before or after 34 weeks (square markers) of gestation. The cyan squares are data from studies that did not distinguish between early versus late preeclampsia and, therefore, combine data for these two groups. RMSE in gray, red, and blue indicate values for normal, early preeclampsia, and late preeclampsia, respectively.

The carotid‐femoral pulse wave velocity (*PWV*) is the gold standard noninvasive metric to assess changes in aortic stiffness. Aortic stiffness and *PWV* increase with age and *PWV* is considered a good composite metric to assess vascular aging and a key, independent risk factor for future cardiovascular events (Laurent et al., 2001). In normal pregnancy, the *PWV* decreases in the first half of pregnancy and increase in the second half (Anastasakis et al., 2008; Callaghan et al., 2018; Everett et al., 2012; Franz et al., 2013; Kaihura et al., 2009; Khalil et al., 2012; Khalil et al., 2014; Mahendru et al., 2014; Mahendru et al., 2017; Rang et al., 2007; Robb et al., 2009; Rodriguez et al., 2018; Savvidou et al., 2011; Tay et al., 2018; van der Graaf et al., 2013). In early and late forms of preeclampsia, *PWV* is elevated by ~1.13–1.18‐fold early in pregnancy, then increases more dramatically as blood pressure increases (Callaghan et al., 2018; Kaihura et al., 2009; Robb et al., 2009) (Figure 5). The carotid‐radial *PWV* also follows this trend (Anastasakis et al., 2008; Kaihura et al., 2009; Khalil et al., 2012; Khalil et al., 2014; Robb et al., 2009; Savvidou et al., 2011).

The augmentation index (${\mathrm{AI}}_{x}$) is another metric used to assess arterial stiffness. ${\mathrm{AI}}_{x}$ is assessed using pulse wave analysis, wherein the blood pressure waveform in the ascending aorta is estimated by applying a generalized transfer function on the blood pressure waveform from a peripheral artery (typically the radial or brachial artery). The aortic blood pressure waveform has two characteristic pressures, $P_{1}$ and $P_{2}$, which represent the systolic peak of the advancing blood pressure waveform ($P_{1}$) and the peak of the composite of the advancing and reflecting waveforms ($P_{2}$). ${\mathrm{AI}}_{x}$ is the ratio of the augmentation pressure ($\mathrm{AP}=P_{2}-P_{1}$) and the pulse pressure (aortic *DBP* – aortic *SBP*). With arterial stiffening, the reflecting wave returns faster and stronger to the ascending aorta, increasing $P_{2}$, thereby increasing AP and ${\mathrm{AI}}_{x}$. Wilkinson et al. also showed that ${\mathrm{AI}}_{x}$ is correlated with heart rate and suggest that, for appropriate comparison of ${\mathrm{AI}}_{x}$ across groups, ${\mathrm{AI}}_{x}$ should be adjusted to a value at a heart rate of 75 beats per minute, denoted ${\mathrm{AI}}_{x}^{75}$ and calculated as ${\mathrm{AI}}_{x}^{75}=0.39\left(\mathrm{HR}-75\right)+{\mathrm{AI}}_{x}$ (Callaghan et al., 2018; Wilkinson et al., 2000). In normal pregnancy, ${\mathrm{AI}}_{x}^{75}$ decreases in the first trimester, then increases or remains nearly constant in the second and third trimester (Kaihura et al., 2009; Khalil et al., 2012; Khalil et al., 2014; Lampinen et al., 2006; Mahendru et al., 2014; Mahendru et al., 2017; Robb et al., 2009; Savvidou et al., 2011; Tay et al., 2018; van der Graaf et al., 2013). With early and late forms of preeclampsia, ${\mathrm{AI}}_{x}^{75}$ is elevated, compared with normal pregnancy, particularly in the third trimester, when blood pressure is elevated (Kaihura et al., 2009; Khalil et al., 2012; Khalil et al., 2014; Robb et al., 2009; Savvidou et al., 2011; Tay et al., 2018).

The in vivo diameter of the ascending aorta increased modestly for the normal and late forms of preeclampsia simulations and remains nearly constant for the early preeclampsia simulation. The in vivo ascending aortic artery diameters are governed by the target wall shear stress and cardiac output. Given the pressure changes throughout gestation, the reference diameters ($d_{\mathrm{ref}}$, defined at 100 mm Hg) decrease as the blood pressure increases. The in vivo distensibility $D\left(P\right)$ decreased in the third trimester in all cases; more stiffening occurred in early and late forms of preeclampsia. However, the reference distensibility $D_{\mathrm{ref}}$ remained nearly constant throughout gestation, suggesting that the large changes in *PWV* and ${\mathrm{AI}}_{x}^{75}$ are mainly attributed to the strain‐stiffening response (Equation 6) and not attributed, significantly, to true changes in material properties associated with a true remodeling response (Equation 10).

## 4 DISCUSSION

This paper presents illustrative 1D FSGR models that describe changes in hemodynamics, vascular geometry, and material properties for early and late forms of preeclampsia and uncomplicated pregnancies; modeling results are compared literature reported values. Early forms of preeclampsia were characterized by a lower cardiac output and elevated total peripheral resistance throughout gestation compared with uncomplicated, normotensive pregnancies. Literature results that distinguish between early versus late forms of preeclampsia are few and findings show a wide range of variability, with early preeclampsia CO = 0.68 to 1.0 MoM. Indeed, our results suggest that there is <5% increase in cardiac output from conception to term in early preeclampsia, compared to a ~ 30% increase in uncomplicated pregnancies. Despite the lower CO, the elevated TPR results in blood pressures that are higher throughout pregnancy, approaching values that are nearly 1.4‐ to 2.0‐fold higher beyond 20 weeks that leads to the pre‐term hypertension. These differences seen with early preeclampsia are coincident with higher values of uterine artery pulsatility index, reaching 1.4‐fold higher at 12 weeks and reaching 2.0‐fold higher values in the third trimester. The differences in TPR may increase later in gestation, compared to the differences in UA‐PI, which may suggest that early preeclampsia may be initiated by impairments in uterine vascular remodeling and elevations in TPR may be a secondary response to increase blood flow to the higher resistant uterine vasculature. Unlike early forms of preeclampsia, late forms were characterized by a slightly (5%–13%) higher cardiac output and nearly identical TPR compared with uncomplicated pregnancies during the first 32 weeks of pregnancy, resulting in a slightly (5%–6%) higher blood pressure. After 32 weeks, TPR and blood pressure increase dramatically, leading to hypertension at term.

These model findings, based on literature results, suggest that early and late preeclampsia occur via distinctly different etiologies and that these different forms may have a distinctly different set of early risk assessment indicators and may require different prevention and treatment strategies. Elevated UA‐PI at 11–14 weeks of gestation has been identified as a key risk indicator for early preeclampsia and fetal growth restriction. Our illustrative results affirm that UA‐PI may be an appropriate indicator for early preeclampsia, but less predictive for late preeclampsia. Little data are available at 12 weeks, specifically for early forms of preeclampsia, but our results suggest that TPR may be ~25% higher in early preeclampsia, compared with both normal pregnancies and late preeclampsia, due to ~18% lower cardiac output and ~6% higher blood pressure. Thus, UA‐PI and TPR may serve as predictive hemodynamic indicators of early preeclampsia. In contrast, it may be more difficult to distinguish between normal pregnancies and late preeclampsia early in gestation. At 12 weeks of gestation, both CO and MAP are slightly elevated (7% and 5%, respectively) in late preeclampsia, with ~2% difference in TPR. UA‐PI is ~10% higher in late preeclampsia, compared with uncomplicated pregnancies at 12 weeks. These small differences (all ≤10%) render prediction of late preeclampsia challenging. With regard to the prevention of preeclampsia, the ASPRE trial clearly showed distinct differences between the effectiveness of low‐dose aspirin in preventing preeclampsia in high‐risk subjects, showing that aspirin reduced incidence of early preeclampsia by 62%, but had no significant reduction in the incidence of late preeclampsia (Rolnik et al., 2017). Finally, our results suggest that arterial stiffness (e.g., cf‐PWV and AIx‐75) may be elevated early in pregnancy and before conception, in both early and late forms of preeclampsia, and may compliment other measured features to assist in risk assessment; however, again, limited data are available in the first half of pregnancy to substantiate this finding. Note that many of the “non‐gravid” results that we included from the literature come from postpartum assessments, which could include sustained, pregnancy‐induced long‐term cardiovascular changes and not be fully representative of preconception values.

While these computational modeling results were validated, as best as possible, with experimental results from the literature, this study has several key limitations. First, given the lack of available data, as in our previous models, we made multiple bold assumptions regarding the structure of the uterine vascular network structure, geometry, and mechanical properties (Gleason Jr. & Sedaghati, 2022). There is a critical need for a more accurate assessment of the uterine vascular network, including numbers of arcuate, radial, and spiral arteries, and tortuosity of uterine and arcuate arteries, and accurate quantification of placental size, location, and number and size of radial arteries that communicate to the placenta. Second, while the main emphasis of this paper is vascular growth and remodeling with early and late forms of preeclampsia, maternal hemodynamics are also closely related to fetal growth restriction. Indeed, UA‐PI may be a more accurate predictor of fetal growth restriction than early onset preeclampsia (Pilalis et al., 2007). We restrict our attention of the current manuscript to preeclampsia, but hope to extending this work to fetal growth restriction in future work.

Third, there were few data in the literature that distinguish between early and late preeclampsia and compare results to uncomplicated pregnancies. In cases where there were wide variations in the literature values across studies, we made our “best guesses” regarding representative values for the early versus late preeclampsia. Fourth, no one study reported a comprehensive set of validation metrics (e.g., CO, heart rate, TPR, UA‐PI, and cf‐PWV); different metrics often came from different studies and cohorts. The metrics are not independent of each other; for example, changes in heart rate affect changes in UA‐PI and relying on data from one set of cohorts for some metrics and another set of cohorts for other metrics may misinform our “best guesses.” This limitation highlights the need for patient‐specific, comprehensive data sets that quantify changes in cardiac function, vessel geometry, mechanical properties, and blood flow rates, and blood pressure pulse waveforms at multiple locations along the vasculature, for uncomplicated, early preeclampsia, and late preeclampsia. Furthermore, for many metrics (CO, TPR, cf‐PWV, AIx‐75, etc.), studies with data collected before 20 weeks of gestation are sparse; there is a pressing need to collect a more comprehensive set of hemodynamic metrics, beyond UA‐PI, early in pregnancy.

In closure, this paper presents illustrative 1D FSGR models that capture the salient differences in hemodynamics and vascular geometry and mechanical properties with early and late preeclampsia, compared with uncomplicated pregnancies. These simulations capture well the characteristic changes in maternal hemodynamics and vascular properties with preeclampsia. Simulation results of the time‐course of numerous clinical measures may provide insight to improve risk assessment of early and late preeclampsia.

## CONFLICT OF INTEREST STATEMENT

The authors have no conflicts of interest to declare.

## ETHICS STATEMENT

This work did not involve any research involving animals or humans.

## Supporting information


Data S1.
